# Supplementary figures and images for: Genome Complexity Browser: Visualization and quantification of genome variability
Source: PLoS Comput Biol. 2020 Oct 9;16(10):e1008222. doi: 10.1371/journal.pcbi.1008222 (PMC7577506; doi:10.1371/journal.pcbi.1008222)

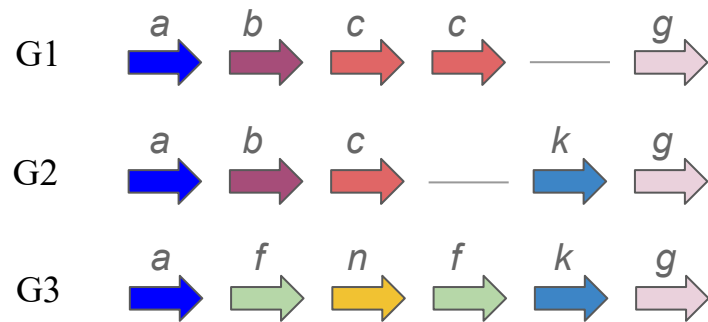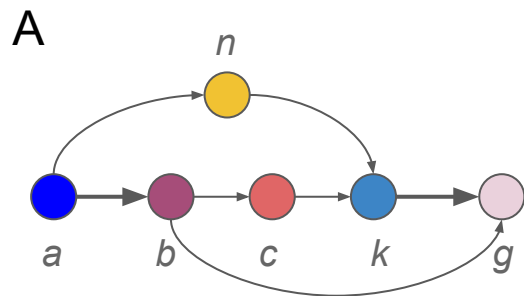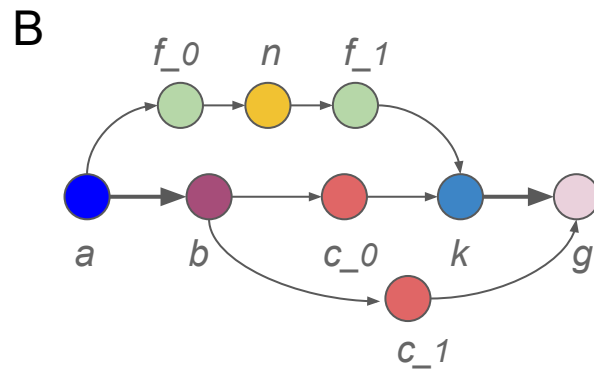

Supplement: S1 Fig — A) The graph obtained with the default approach, which ignores groups with several representatives in the particular genome. B) The graph obtained using the paralog “orthologization” approach. (PDF) [file pcbi.1008222.s001.pdf]

## A hemin uptake operon

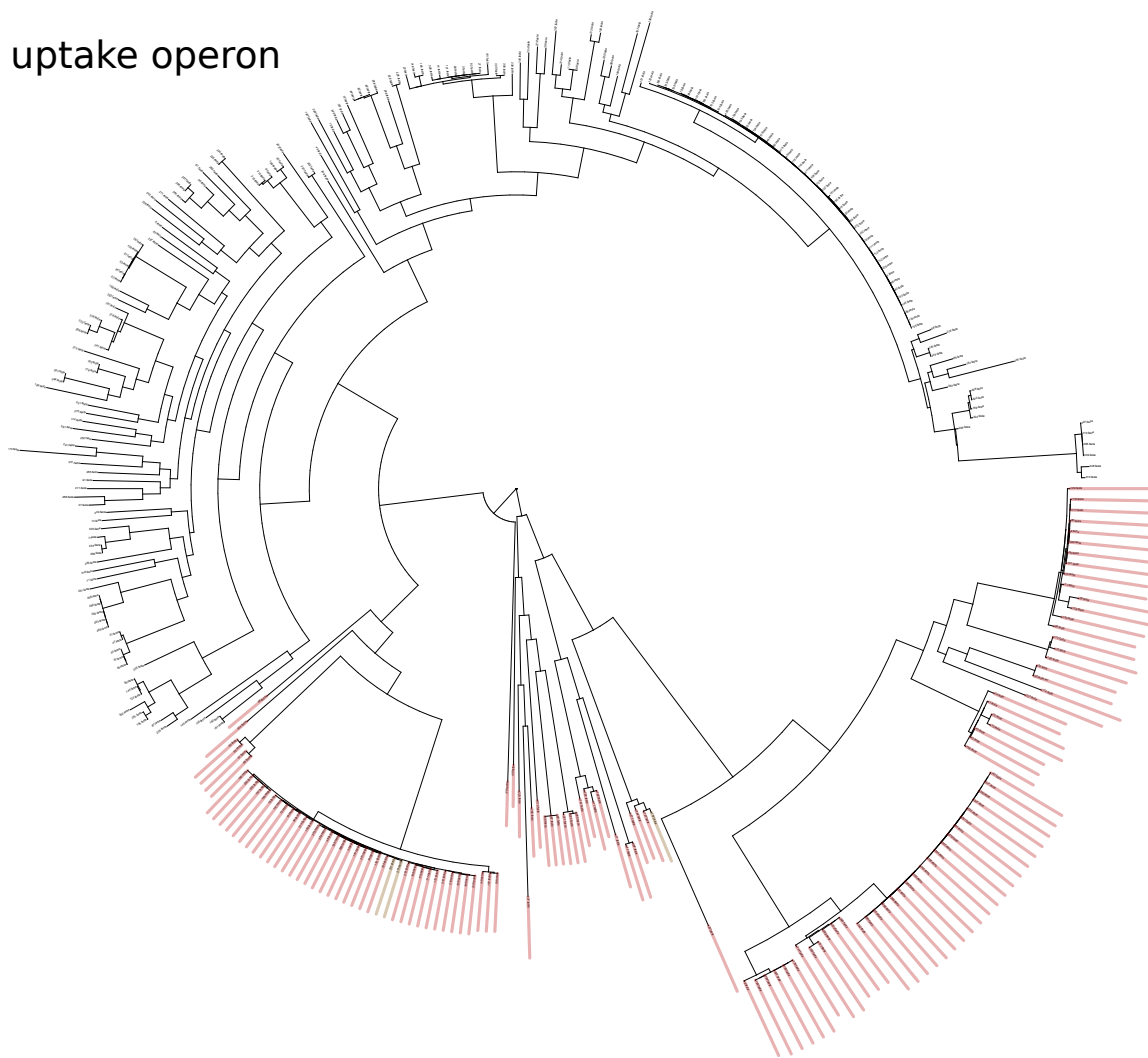

**B** propanediol utilization operon

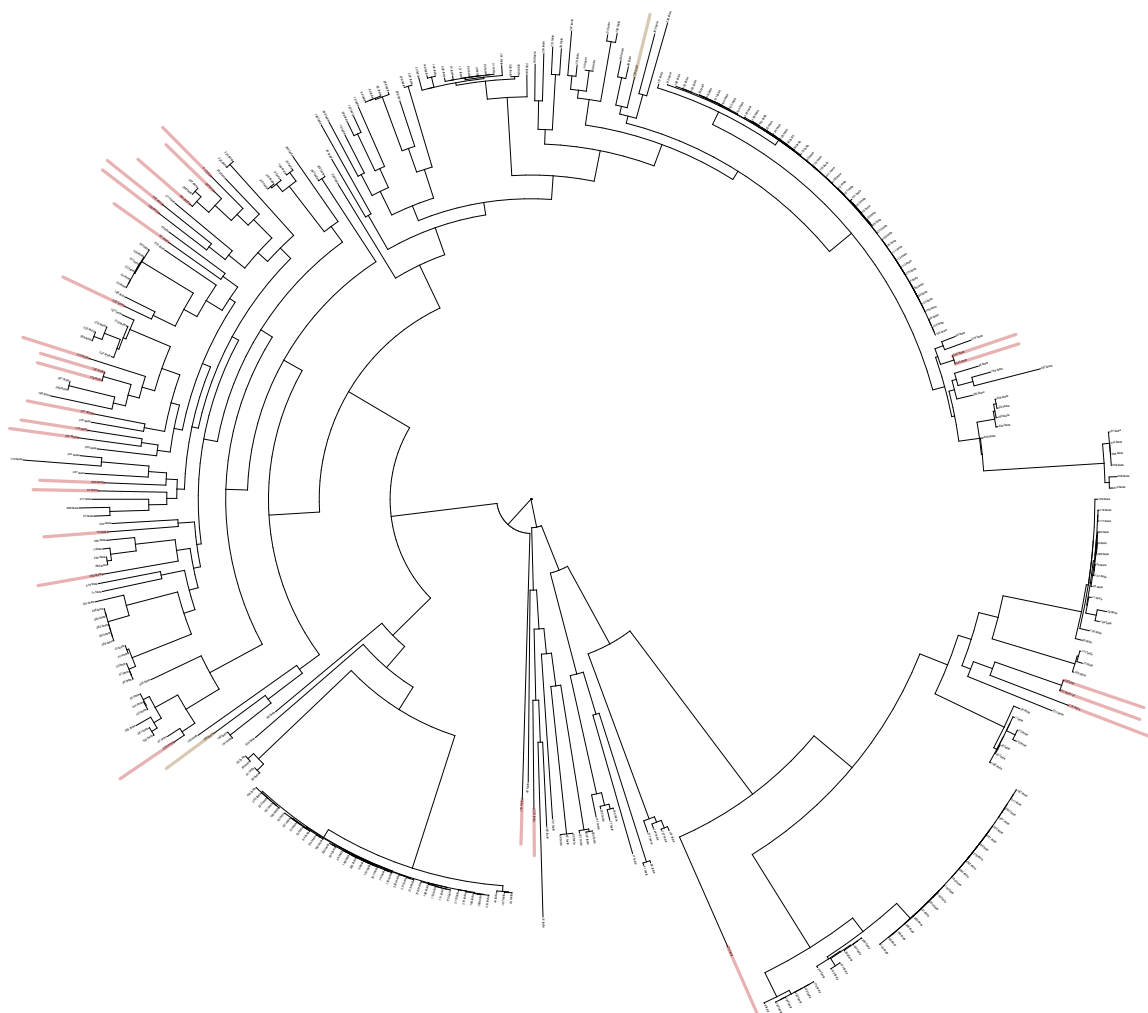

Supplement: S2 Fig — Red bars denote genomes in which the complete gene of the operons is present, green bars denote genomes in which more than half of the operon genes are present. A) The hmu operon is in good correspondence with the phylogenetic tree of E. coli. B) The pdu operon presence is poorly correlated with the phylogenetic tree of E. coli. (PDF) [file pcbi.1008222.s002.pdf]

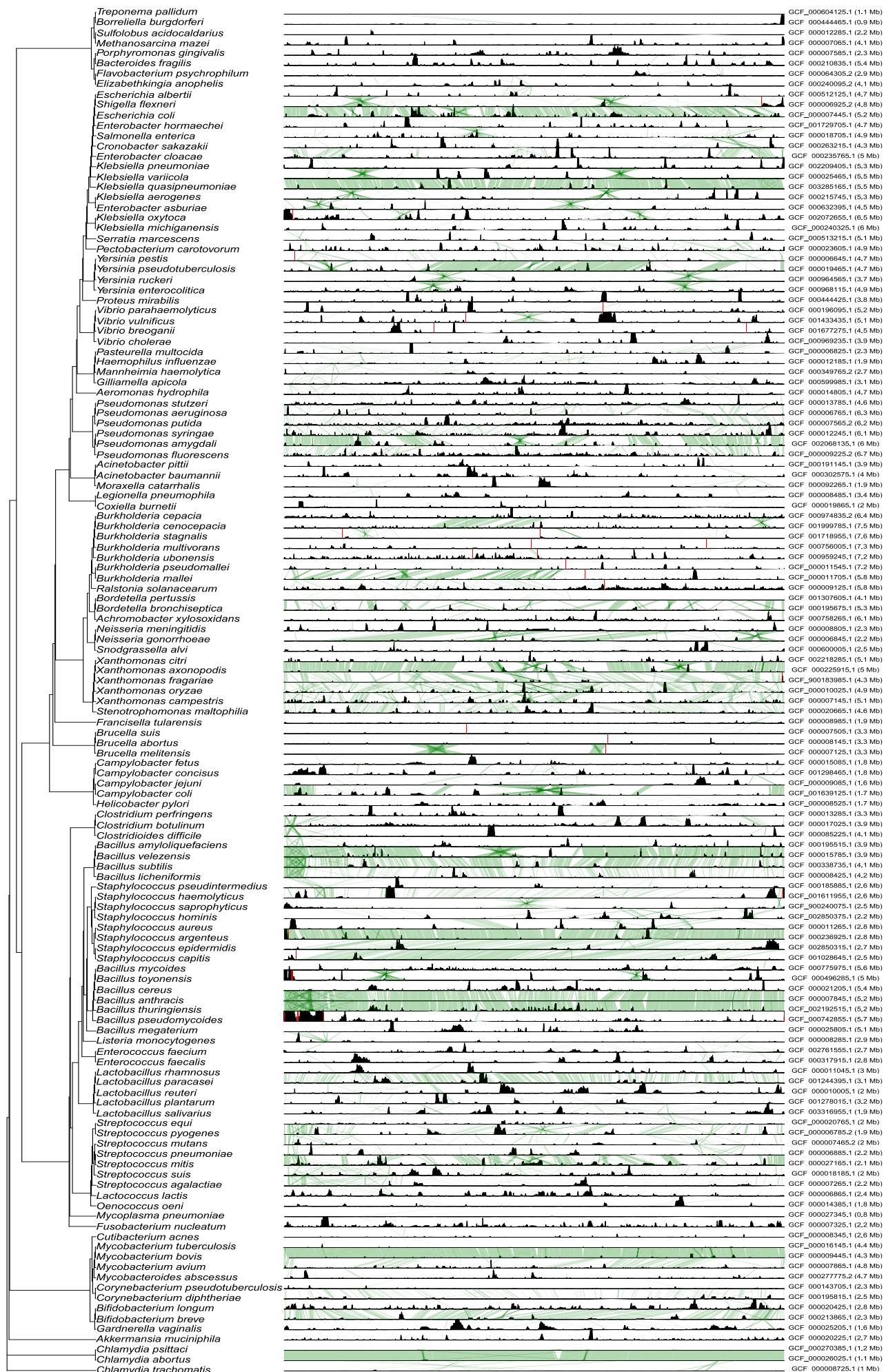

Supplement: S3 Fig — Complexity profiles are shown on the same scale for all organisms. Synteny blocks are shown in green. The phylogenetic tree was built based on the 16S rRNA sequence. (PDF) [file pcbi.1008222.s003.pdf]

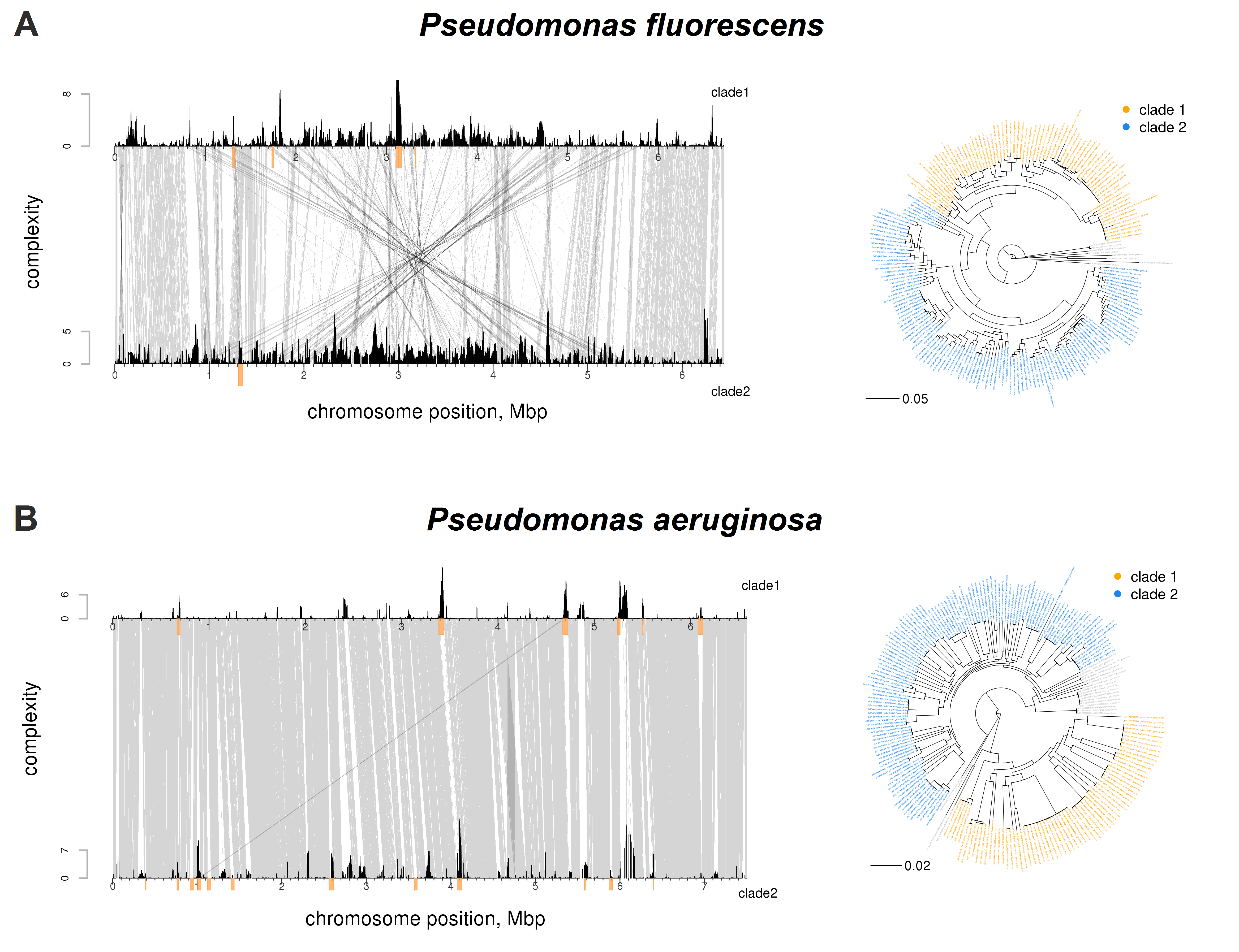

Supplement: S4 Fig — Complexity profiles and synteny blocks are shown on the left, phylogenetic trees are shown on the right. (TIF) [file pcbi.1008222.s004.tif]
