# Supplementary material for: Genome Complexity Browser: Visualization and quantification of genome variability
Source: PLoS Comput Biol. 2020 Oct 9;16(10):e1008222. doi: 10.1371/journal.pcbi.1008222 (PMC7577506; doi:10.1371/journal.pcbi.1008222)
Supplement: S1 Text — Details concerning the methods and results of genome simulations, with predefined variability profiles, and further complexity analysis. (PDF) [file pcbi.1008222.s005.pdf]

Genome variability simulation

To validate our method we assumed non-random distribution of the probability of genome rearrangement events in different genome locations. First, we created 100 identical genomes and then run random genome rearrangements simulations with 3000 iterations. We used three predefined patterns (sinus, sawtooth, rectangular) of the rearrangements probability distribution (Fig 1) through the genome.

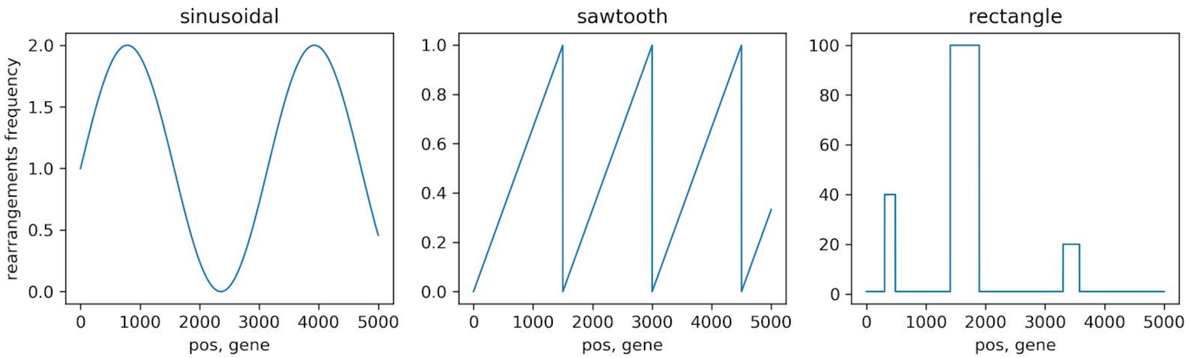

Figure 1. Pre-defined distribution: sinusoidal, sawtooth, rectangular

For each iteration, we performed genome changes: insertion of a new gene(s) (from “orbital” genes set), horizontal gene transfer (HGT) from other genomes, deletion of the gene(s), inversion of part of the genome. The length of each rearrangement was determined by an exponential distribution with $1/\boldsymbol{\lambda}$ = 0.005. HGT and random insertion probabilities were chosen to be equal to deletion events probability to maintain genome length. The probability of inversion was chosen as 1/100 than others, in agreement with literature data that inversion events are less common than other types of rearrangements, such as deletions and duplication [1, 2]. For each distribution type, 10 independent simulations were performed. After all iterations, generated genomes were used to generate gene context graph and our algorithm of complexity estimation was applied and compared to the initial rearrangement distribution (Fig 4C, main text).

Simulations parameters:
insertion probability = 0.5
HGT probability = 0.5
inversion probability = 0.005
genome length = 5000
number of genomes = 100
number of iterations = 3000

To estimate the similarity between input and output distributions R-square value and Spearman correlation coefficient was used (Table 1). FDR Benjamini-Hochberg multitest correction was applied to correct p-values of these tests for each model. In all cases calculated p-values were smaller than floating-point type numbers threshold ($< 10^{-308}$).

Table 1. Simulation results

simulation	r-square	Linregress p-value	spearman correlation coefficient	Spearman test p-value
sin0	0.747845297872213	$< 10^{-308}$	0.8087390103892563	$< 10^{-308}$
triangle0	0.7863740580066408	$< 10^{-308}$	0.8407506199412642	$< 10^{-308}$
rectangle0	0.9517350270068695	$< 10^{-308}$	0.6956340770043501	$< 10^{-308}$
sin1	0.773356469186511	$< 10^{-308}$	0.8223263631263291	$< 10^{-308}$
triangle1	0.826142489530444	$< 10^{-308}$	0.850815752496576	$< 10^{-308}$
rectangle1	0.9384762081932699	$< 10^{-308}$	0.6944633178815065	$< 10^{-308}$
sin2	0.8020718309173395	$< 10^{-308}$	0.8570654755007394	$< 10^{-308}$
triangle2	0.7688398470945833	$< 10^{-308}$	0.8385260939693582	$< 10^{-308}$
rectangle2	0.9498992345534695	$< 10^{-308}$	0.6911063423010434	$< 10^{-308}$
sin3	0.7435280319501681	$< 10^{-308}$	0.8214729109148974	$< 10^{-308}$
triangle3	0.81089688751292	$< 10^{-308}$	0.8501003339066999	$< 10^{-308}$
rectangle3	0.9444214653118793	$< 10^{-308}$	0.7295408855212339	$< 10^{-308}$
sin4	0.7570741401402435	$< 10^{-308}$	0.8124578904045474	$< 10^{-308}$
triangle4	0.7900341396135132	$< 10^{-308}$	0.846736477776503	$< 10^{-308}$
rectangle4	0.9535867432304211	$< 10^{-308}$	0.6998598673455297	$< 10^{-308}$
sin5	0.7624979912000555	$< 10^{-308}$	0.8153347347081705	$< 10^{-308}$
triangle5	0.8018717359719779	$< 10^{-308}$	0.8448690068735133	$< 10^{-308}$
rectangle5	0.9306747376737399	$< 10^{-308}$	0.6971589300503787	$< 10^{-308}$
sin6	0.7738466227491204	$< 10^{-308}$	0.819660030578236	$< 10^{-308}$
triangle6	0.8105119088229455	$< 10^{-308}$	0.8476708448368908	$< 10^{-308}$
rectangle6	0.9609338711203066	$< 10^{-308}$	0.6943738005422859	$< 10^{-308}$
sin7	0.7798603141004977	$< 10^{-308}$	0.82695388256868	$< 10^{-308}$
triangle7	0.7891722253984607	$< 10^{-308}$	0.8426963862172815	$< 10^{-308}$
rectangle7	0.9544905829553292	$< 10^{-308}$	0.6977930247944066	$< 10^{-308}$
sin8	0.7881324306446891	$< 10^{-308}$	0.8424745671486148	$< 10^{-308}$
triangle8	0.8105059194018976	$< 10^{-308}$	0.8525784149425557	$< 10^{-308}$
rectangle8	0.9464876860171973	$< 10^{-308}$	0.6938931858082366	$< 10^{-308}$
sin9	0.7434002936999184	$< 10^{-308}$	0.7911308855786456	$< 10^{-308}$

triangle9	0.8070283911154931	$< 10^{-308}$	0.8511579011145844	$< 10^{-308}$
rectangle9	0.9544292820433158	$< 10^{-308}$	0.6965411860311865	$< 10^{-308}$

It is possible to carry out the validation pipeline with one sinusoidal distribution by a script that is available on http://github.com//DNKonanov/geneGraph/. Validation script "validate.sh" is located in "geneGraph-master/source/validation/". This short pipeline includes random rearrangements simulations, complexity computing and results plotting.

References

1. Anderson, R. P., and J. R. Roth. Tandem genetic duplications in phage and bacteria. Annu. Rev. Microbiol. 1977; 31:473-505.
2. Schmid, M. B., and J. R. Roth. Selection and end point distribution of bacterial inversion mutations. Genetics. 1983;105:539-557
