# Supplementary material for: Genome Complexity Browser: Visualization and quantification of genome variability
Source: PLoS Comput Biol. 2020 Oct 9;16(10):e1008222. doi: 10.1371/journal.pcbi.1008222 (PMC7577506; doi:10.1371/journal.pcbi.1008222)
Supplement: S2 Text — MCC, F1 score and precision values are given. (PDF) [file pcbi.1008222.s006.pdf]

Comparison of hotspots identified by GCB with a curated dataset.

Genome complexity estimating method was assessed as the way for genomic islands (GI) detection. We assumed that regions with high complexity values (hotspots) may represent genomic islands. To detect hotspot regions we used common outlier detection method proposed by Tukey:

$$complexity_i >= Q3 + 1.5*(Q3-Q1),$$

where *Q1* and *Q3* are 0.25 and 0.75 percentiles of complexity profile. Hotspots regions were regarded as GIs.

*

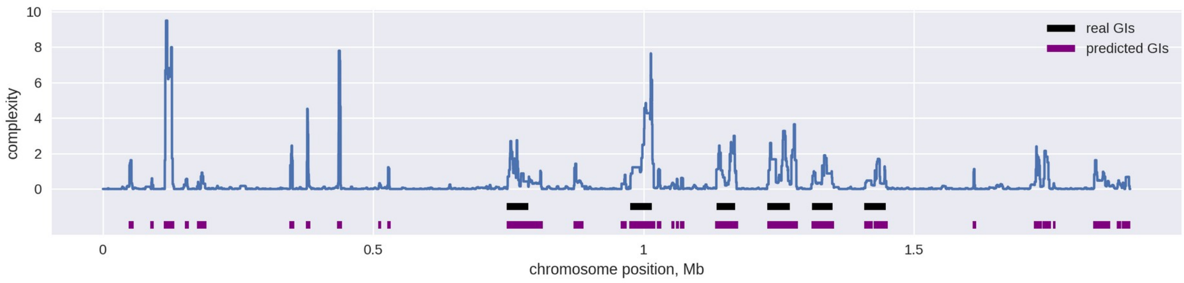

Fig 1. Example of complexity based approach for prediction genomic islands in *Streptococcus pyogenes* MGAS315, NC_004070.1 chromosome.

To get a confusion matrix trueness of the prediction was checked for each nucleotide separately. MCC, F1 score and precision metrics were used to estimate quality of GI prediction:

$$MCC = \frac{TP*TN - FP*FN}{\sqrt{(TP+FP)(TP+FN)(TN+FP)(TN+FN)}}$$

$$F1score = \frac{2TP}{2TP+FP+FN}$$

$$precision = \frac{TP}{TP+FP}$$

From the genomes available in curated literature-based dataset [1] only 4 chromosomes were also present in our precalculated set.

Results:

chromosome	MCC score	F1 score	precision
NC_004431.1	0.6398974787389852	0.6942167816826945	0.6039911283491597
NC_004070.1	0.655472809862469	0.6844523104895646	0.5494813503524955
NC_003923.1	0.5952671253837918	0.5892016756020617	0.4286045699930403

NC_002695.1	0.5469882821186525	0.622686519552352	0.5896994002603333
**mean**	0.609406424025975	0.647639321831668	0.542944112238757

By MCC and F1-score this approach is comparable to existing tools such as IslandPath-DIMOB, GIHunter and IslandViewer 4. But there is a huge number of false positive detected GIs, so, the precision score is very low compared to other methods.

References dataset obtained by using the comparative genomics approach of IslandPick [1], also was used to validate complexity based approach.

chromosome	MCC score	F1 score	precision
NC_009708.1	0.38487104690031365	0.3734107527432849	0.2451605459656494
NC_007005.1	0.2054826380489073	0.25008881198628097	0.17050550994785096
NC_010501.1	0.1656805277399133	0.2497633887301143	0.22984014707278763
NC_008253.1	0.31920178349975015	0.3263252331260368	0.21389808518495657
NC_009076.1	0.238251125314444	0.2786501010119271	0.18458251701690434
NC_009487.1	0.0903831957409384	0.05332905771389135	0.02803553366462905
NC_004578.1	0.25403435531999186	0.34446613497841755	0.286251319388785
NC_008463.1	0.30182788811643274	0.2992938488723532	0.19363758817684576
NC_003485.1	0.2579527628894837	0.1740849990936506	0.09638635877810856
NC_009512.1	0.27820951615033646	0.3445637427241827	0.3388378919734103
NC_004606.1	0.15200885258105978	0.10312124791584637	0.05589103172094004
NC_010167.1	0.000466314090059021	0.12570593895109675	0.33321936163527827
NC_007530.2	0.06398331158846338	0.04689342484723675	0.024840549813711664
NC_010410.1	0.3766808133470356	0.2920455742723105	0.17116491420042124
NC_005139.1	0.19703389818572514	0.150013006485706	0.08424900608332814
NC_008061.1	0.19022392344977773	0.3225759083357807	0.33217554977792224
NC_010084.1	0.253070606088803	0.26344416769198453	0.16848999568158918
NC_003997.3	0.08390639743001588	0.05575501749959223	0.029555952338787134
NC_009080.1	0.12260548261530846	0.14317482232583406	0.0870655839240245
NC_010515.1	0.04983208533937494	0.2227876295579963	0.24346152492483925

NC_000907.1	0.09296002068782915	0.034584556473813956	0.017713963421374095
NC_008563.1	0.3935844374822941	0.38242186952979623	0.2515840311452063
NC_009800.1	0.19061720695979295	0.09702586444736065	0.051312273685759746
NC_010498.1	0.327133637346465	0.2597803467991166	0.15236272552588775
NC_004088.1	0.05911317631934262	0.052376147229830375	0.02926301686490688
NC_004116.1	0.2495949994749722	0.17778414841262602	0.09951760827068401
NC_008060.1	0.3415246066690782	0.44232779482012574	0.37481743033385473
NC_002944.2	0.07018640090967038	0.07504233105244104	0.042077179321005555
NC_010582.1	0.13071463422847124	0.12897879089527856	0.07521532748386954
NC_004368.1	0.28484822356765815	0.31553935338230515	0.21422814331310497
NC_007146.2	0.36024382180513154	0.3149167421444994	0.193772219653427
NC_010380.1	0.2336884661910294	0.23388640964119076	0.14489939693845189
NC_007434.1	0.3833920912799913	0.45966224818025575	0.35400710325841206
NC_004431.1	0.47005108656504924	0.5091760136438895	0.37988349456852866
NC_010465.1	0.37337575395475303	0.36600955692191756	0.23925061631367175
NC_006350.1	0.3458290477930752	0.39563460781220766	0.28059620614261555
NC_010473.1	0.06707619633431747	0.044255182787441144	0.023417902855171528
NC_002695.1	0.3903822256193024	0.46597765476482533	0.38414359689708727
NC_004070.1	0.2367698334411089	0.131330169255604	0.07032568299222543
NC_010102.1	0.2814133285495277	0.22346937518731244	0.12993039443155452
NC_008543.1	0.2128452820810379	0.2672648562640155	0.1898330414767529
NC_010184.1	0.1008380525664982	0.05916659388971843	0.03125161731648099
NC_008600.1	0.03806952653775363	0.022079794650779565	0.01136477165453867
NC_006155.1	0.3077655936920609	0.29831291471034593	0.18815448858629918
NC_008595.1	0.2322583123376348	0.29932392244640826	0.21834619883281148
NC_003909.8	0.18936903106619443	0.13706508158499245	0.076058265611407
NC_010159.1	-0.03414126616396267	0.003108575879328839	0.001715206630597444
NC_008022.1	0.19154922127994686	0.17830576368746082	0.10471699362854835
NC_002516.2	0.08692207405991095	0.08808184125921077	0.04971620468391139
NC_007946.1	0.46096590075508376	0.5136607961327568	0.40380701925813894

NC_003923.1	0.18794417019300338	0.09020057602747758	0.04742209004136109
NC_010322.1	0.2321622659018411	0.3050790907253817	0.3218192076660855
NC_008024.1	0.31654162612791337	0.21890083701594967	0.12290210537699855
NC_007432.1	0.33099343383472785	0.2967752098476495	0.18335437371046587
NC_009801.1	0.4078800664945426	0.3496928952424733	0.2142028994220997
NC_004722.1	0.16287408707503673	0.08902883035739562	0.04719050652395732
NC_005945.1	0.07993009039643954	0.055643426639708664	0.029571384905213122
**mean**	0.22408724890966067	0.2245853154141476	0.16257883607040766

References

1. Bertelli C, Tilley KE, Brinkman FS. Microbial genomic island discovery, visualization and analysis. Briefings in bioinformatics. 2019;20(5):1685–1698.
