# Supplementary material for: Genome Complexity Browser: Visualization and quantification of genome variability
Source: PLoS Comput Biol. 2020 Oct 9;16(10):e1008222. doi: 10.1371/journal.pcbi.1008222 (PMC7577506; doi:10.1371/journal.pcbi.1008222)
Supplement: S1 Listing — (PDF) [file pcbi.1008222.s007.pdf]

SUPPLEMENTARY LISTING 1. ALIGN GENOMES SET

Input: non-reversed nodes chains
Output: list of aligned genomes

all_contigs ← get full contigs list from non-reversed chains
sort all_contigs by their length

aligned_contigs ← empty list

while all_contig is **not** empty **do**

reference_contig ← longest (first) contig from all_contigs

for each contig **in** all_contigs **do**
if contig is reference_contig **do**
move contig from all_contig to aligned_contigs

if reference_contig do not contains at least 50% of nodes from contig **do**
continue

forward_count ← count of pair of sequently located gene contained in both reference_contig and contig
reverse_count ← count of pair of sequently located gene contained in both reversed reference_contig and contig

//forward_count and reversed_count calculated with multiplication by the length of the corresponding genes

if reverese_count > forward_count **do**
move reversed contig from all_contigs to aligned_contigs

else do
move contig from all_contigs to aligned_contig

list of aligned genomes ←sort aligned contigs by organisms
return list of aligned genomes
