# Supplementary material for: Genome Complexity Browser: Visualization and quantification of genome variability
Source: PLoS Comput Biol. 2020 Oct 9;16(10):e1008222. doi: 10.1371/journal.pcbi.1008222 (PMC7577506; doi:10.1371/journal.pcbi.1008222)
Supplement: S2 Listing — (PDF) [file pcbi.1008222.s008.pdf]

SUPPLEMENTARY LISTING. GENOME VARIABILITY SIMULATION

Input: changes frequency distribution, probability of different types of changes (HGT, deletion, inversion), parameters
Output: graph.sif file with simulated genomes set

```
genomes ← set of identical genomes with 5000 genes
i ← 0
while i < number of iterations do
        genome ← random genome from genomes
        //random(0,1) generates random float number between 0 and 1
        if random(0,1) <= inversion_probability do
                inversion_length ← random value from exponential distribution with 1/λ = input exponential parameter
                inversion_position ← randomly generated position in genome based on input distribution
                apply inversion

        if random(0,1) <= insertion_probability do
                insertion_length ← random value from exponential distribution with 1/λ = input exponential parameter
                insertion_position ← randomly generated position in genome based on input distribution
                inserton_chain ← random list of "orbital" genes with length insertion_length
                // "orbital" genes is set of genes named from 5000 to 5000+number of "orbital" genes
                apply insertion
                apply random deletion //It's necessary because we need to store the same number genes in genomes

        if random(0,1) <= HGT_probability do
                other_genome genome ← random other genome from genomes
                HT_length ← random value from exponential distribution with 1/λ = input exponential parameter
                HT_position_from ← randomly generated position in genome based on input distribution
                HT_position_to ← randomly generated position in other_genome based on input distribution
                apply HT
                apply random deletion from other_genome
        i ← i + 1
write genomes to *.sif file
// organism are named as org0, org1, …, orgN and org_ref, where org_ref is no-changed chain (from 0 to 4999)
return
```
