# Supplementary material for: Genome Complexity Browser: Visualization and quantification of genome variability
Source: PLoS Comput Biol. 2020 Oct 9;16(10):e1008222. doi: 10.1371/journal.pcbi.1008222 (PMC7577506; doi:10.1371/journal.pcbi.1008222)
Supplement: S3 Listing — (PDF) [file pcbi.1008222.s009.pdf]

SUPPLEMENTARY LISTING. GENERATE SUBGRAPH

Input: graph, reference, start_node, end_node, window, depth, tails, minimal_edge_weight
Output: subgraph

subgraph ← empty graph

target_chain ←nodes chain from reference genome between (start_node - window) and (end_node + window)

add base_chain to subgraph

deviating_paths ← find all deviating paths connected with target_chain

for each path **in** deviating_paths **do**
    if length of the path <= depth **do**
        add path to the subgraph
    else do
        path_tails ←left and right fragments of the path with length *tails*
        add path_tails to the subgraph

for each edge **in** subgraph **do**
    if edge weight < *minimal_edge_weght* **do**
        delete edge from the subgraph

clear the subgraph from disconnected fields

return subgraph
