# Supplementary material for: Genome Complexity Browser: Visualization and quantification of genome variability
Source: PLoS Comput Biol. 2020 Oct 9;16(10):e1008222. doi: 10.1371/journal.pcbi.1008222 (PMC7577506; doi:10.1371/journal.pcbi.1008222)
Supplement: Attachment — Submitted filename: response.pdf [file pcbi.1008222.s011.pdf]

Reviewer #1: In this study Manolov et al propose the 'Genome Complexity Browser' (GCB), a novel tool allowing visualization of gene contexts in a graph form and quantification of genome variability.

The manuscript is clear, relatively well-written, and fills a gap in the sense that it provides a relatively accurate measure of gene variability at a local level (by computing the complexity value).

I have the following observations / criticisms, which I hope will help the authors improve their manuscript:

1) The authors mention multiple times 'hotspots' as chromosomal loci in which HGT events are preferentially concentrated. In what measure (particularly via the complexity value), does GCB helps disentangling between regions of the chromosome that are (or are not) hotspots? Although valid, the complexity value computed by the authors will only be useful for the wide community if it is able to provide some clue about what constitutes a hotspot. In other words, above which threshold values of complexity can we call a region a hotspot?

- We agree that it would be convenient for a user to get hotspot regions immediately, without additional analysis. We have added hotspot identification features, both for web- and a stand-alone version. We tried several methods of hotspot identification (peak identification, percentile approach) and ended with the interquartile method of Tukey, as it was the most robust one. Corresponding text was added to the Methods section. Users may change the value of coefficient to get more or less stringent hotspots.

2) The complexity values computed by the authors, also do not provide per se any information about the types of rearrangements taking place. For this, the user must analyze the graph representation. While the latter is useful for a trained user, it won't be for many others. In this sense, it would be useful to split the complexity value into terms corresponding to replacements, gains, losses, etc. This would give the user a more immediate sense (and some quantification as well) of the frequency of these processes. It would also provide an idea if the region is, for example, subjected to high gene turnover, or is undergoing differential gene loss.

- We observed that the HGT hotspots in genomes appear as the result of multiple overlapping events, both deletions and insertions. While phylogenetic analysis and reconstruction of ancestral states is indeed an interesting field of further research, for now, we think that the main goal of the GCB is to provide information about genome variability. Variability profile may be used in comparisons and other types of downstream analysis, according to user tasks.

3) I presume that all the orthologous computed by the authors are positional ones? How does GCB handle, for example, genomic inversions?

- We compute orthogroups based on proteins reciprocal blast scores, without use of a positional information. Positional information is used in the graph construction step and in paralogous

genes orthologization step. In the graph, genomic inversion will result in addition of two long edges and edges of neighboring genes, inside the inverted region of the genome, will be connected in the opposite direction (these edges may be hidden with the use of "Hide reversed" option in the Settings panel).

4) Discussion: “On the other hand, it doesn't take into account phylogenetic information and syntenic relationships between different genomes, and erroneous homology inference sometimes occurs.” How often did this happen? The authors need to show some numbers. How many times paralogous genes were attributed to one group?

- We have added information on the frequency of paralogs: average values for all species in the main text, and values for individual species in an supplementary table. 0.5% of all orthogroups per genome contain at least one paralogous gene; among all orthogroups inferred for the species, the proportion of orthogroups with paralogs is almost 16%. This is the average values for all considered species, values for each species are shown in the S1 Table.

5) “Quantitative estimation of genome variability along the chromosome” -> “Quantitative estimation of variability along the chromosome”

- Fixed. Thank you for the remark.

****Reviewer #2:** Summary**

The authors present here a novel tool entitled the Genome Complexity Browser to infer and visualize hotspots of genome variability across many prokaryotic genomes. The subject is of large interest to a broad audience and the software surely fills a gap in available tools for genome comparison. There are wide possible applications for this type of analyses, that are briefly covered in the result section. However, the manuscript suffers from the quality of the reporting and many aspects need to be clarified.

Major comments

Introduction

The first paragraph of the introduction should be improved to better explain the concept of genome architecture and constraints ruling gene order and genome rearrangements. “Regularities” is likely not the appropriate word here.

- We modified the first paragraph and referred to the description of genome architecture from [Rocha EP. The organization of the bacterial genome. Annual review of genetics. 2008].

Materials and methods

The section misses a general description of the algorithm, eventually with a schematic figure, to allow a better understanding of the standalone tool: input, steps computed by the tool, output, visualization? Also, the authors must allow the reader to understand immediately the differences between the standalone tool and the webserver, including the type of available data.

- We have added a roadmap of the standalone analysis (Figure 2B). For greater ease of use, all logic was combined into two main pipelines. Information regarding differences of standalone tools and the webserver was added to the Methods section.

The authors recommend that contigs of draft genomes are re-ordered to increase co-linearity before using their tools. What is the influence of having draft genomes on the accuracy of results obtained? It would be interesting to benchmark this aspect by comparing the results of a small set of complete vs draft genomes or reordered vs non-reordered genomes, highlighting and discussing potential biases.

- We have added results of GCB benchmark to the Results section (Method applicability, Figure 7). Our comparison showed that the coalign step, which increases co-linearity of contigs, slows down the analysis, when many draft genomes are used, and practically does not affect the complexity values (Figure 7 C,D); we have made this step optionally. Draft genomes may be used for the analysis without significant impact on the complexity values (Figure 7 A), but will lead to a lack of some edges in a subgraph visualizations. Discussion was added to the Method applicability section.

Results

Software description and availability:

The descriptions should be improved on all levels in the manuscript, with appropriate links provided, e.g. to the Snakemake script to infer homology groups (name, place?). The wording seems to imply that it can be used to identify homology groups, which could be understood as building groups of orthologs but does not seem to be the case. Please clarify.

- We have added descriptions and links to all used scripts and pipelines to the text. Term "orthogroup" is now used instead of "orthology group" or "homology group" throughout the text. Orthogroups may contain paralogs. These paralogs can be orthologized based on neighboring genes (a gene in each of the unique contexts becomes a separate node) when constructing a graph.

“GCB browser-based GUI consists of three main parts”. Actually, there are four panels with the search panel. It should be described as well to respect the order in figure 2. Numbering the panels on the figure 2 would also improve reader's understanding at first sight.

- The GUI has been changed; we tried to make it more accurate and clear. Now, all settings are available in the left sliding panel. Description was modified and panels on Figure 2 were numbered.

Finally, a more detailed description of possible options in the manuscript (e.g. loading custom features to display) and on the website would be useful to the users (e.g. use and file format to input with “load file”).

- Descriptions of most oftenly used options were added to the manuscript now.

The overall look of the webserver is disappointing, and common information for such tools are not available, such as FAQ, address/way to contact the developers or maintainers, etc.

- Contact information and link to the documentation are available in the About tab of the left slide panel now. Direct links to documentation have been added to the manuscript.

The display of panel 1 is quite confusing. For example, if one fills OG, then coordinates must be updated (but the button appears a few lines below) and if one fills the coordinates, then OG must be updated (but the button is above). I would suggest revising the position of the different items so that users are directed on the correct button after filling the various fields.

- We modified GUI. Now OG are not used to indicate regions of the genome, so as not to confuse users (users are unlikely to know the required OG, but can find information on the coordinates of the regions of interest to them). We implemented automatic positioning for graph drawing.

The authors give many examples of how their tools could be useful to study variations in genome architecture within and among species. However, the description of examples and their corresponding figures is often very superficial, and leaves the reader with many questions. For example, how does the tool perform depending on the distance between genomes compared? As shown in some examples, it works well with highly collinear genomes. What is the effect of recombination prone bacterial species such as Pseudomonas on the assessment of genome variability? Can the tool be used efficiently to study more distantly related organisms, and under which conditions?

- Complexity analysis is intended to evaluate local genome variability. The applicability of the method is limited to cases where local variability is present and is not obscured by large-scale changes in the genome. This is not a very clear boundary, but we can suggest (based on our experience and estimates from [Brilli, Matteo, et al. "Short and long-term genome stability analysis of prokaryotic genomes." BMC genomics (2013)]) that complexity values should be calculated based on the genomes belonging to the same species or intraspecific structures. The discussion of this point was added to the Method applicability section.
We performed comparison of complexity profiles of intraspecies structures for two Pseudomonas species (P. aeruginosa and P. fluorescens). The complexity profile for naturally

competent P. fluorescens was relatively uniform (which is not typical for most of the species considered); complexity profiles of two different phylogenetic clades revealed little similarity, also one complexity peak in a conserved neighbourhood was clearly visible (S4_Fig). This may be due to significant genome rearrangements. The opposite is observed for the P. aeruginosa. Complexity profiles for these species had clear peaks; comparison of complexity profiles of different phylogenetic clades revealed significant similarity (S4_Fig).
Complexity profiles of distantly related organisms may be compared, but this comparison will be informative only if the genomes of these organisms possess syntenic regions, then conclusions can be made regarding change in the activity of hotspots.

The authors elude to the possible use of their tool to identify prophages and genomic islands. Did they try to benchmark their tool with existing reference dataset such as that originally published by Langille et al (2008) and refined in Bertelli et al (2019)? This could be valuable to assess the usefulness of GCB compared to other existing tools.

- We have performed a comparison of complexity hotspots identified by GCB and genomic islands from [Bertelli et al, 2019], the results are added to the Method applicability section and S2 Text. On the curated, literature-based part of the dataset, GCB showed an F1 score of 0.65, which is comparable to existing tools, such as IslandPath-DIMOB, GIHunter and IslandViewer 4. Comparisons with the automatically generated part of the dataset results in much lower accuracy scores. We think that GCB should not be used as a method of choice, when the task is to identify genome islands and prophages (it does not outperforms specialized software), but GCB will be useful to verify findings from other tools, and obtain additional information regarding variability and observed multiplicity of genomic contexts of genome islands or prophages.

Based on the github repository, the software seems to depend on an old version of OrthoFinder. Are the outputs of newer versions supported too? Also, the authors discuss that OrthoFinder does not take into account phylogenetic information, which contradicts the latest publication of OrthoFinder by Emms and Kelly (2019). Please be more accurate in the discussion.

- GCB works well with the output of OrthoFinder, currently available at bioconda (v2.3.12). Indeed, OrthoFinder can use phylogenetic information to infer orthologues now, we removed the mentioned remark from the text. Thank you for noticing the inaccuracy.

Overall comments

The quality of written English must be improved throughout the manuscript as it contains many typo, grammar errors, incorrect words. For example, in “variable and conservative parts” the authors probably mean conserved, or “or it close homologs are present” (its), or simply poorly written sentences “Finally, integrons have expectedly high complexity values computed with here proposed method.”. The authors must make an extensive effort to improve the reporting style and the storytelling.

- We have revised the text, and the manuscript was processed using a professional language editing service.

Minor comments

- “depending on the chromosome position” – I doubt the author mean the position of the chromosome, but rather the position of the gene on the chromosome.

- “ Local genome variability is estimated using a graph representation of gene order (neighborhood) with a here introduced measure called complexity.” Revise English

- Be careful of typing errors such as: “selection f a genome”

- Thank you for a thorough review, we have rewritten these sentences.

****Reviewer #3:** From what I can see Genome Complexity Browser is a visualization tool** showing genome plasticity for complete genome (i.e. from refseq it seems). The concept is good, I think we need to move away from all-against-all comparisons like act, easyfig, mauve (because they wont cope with large datasets). And admittedly BRIG being reference-centric doesn't show the variability very well. AS far as I know there isn't a tool that does this out of the box (sure you can do a combination of BLAST, R, some networking library to get the same effect) so GCB addresses a clear need.

All in all my main criticism for this manuscript and the tool is that both do not help me, as a user, use it. There is a lot of information that is assumed. It is clear that the authors have not given this program to their colleagues to play around it. I suggest strongly that they do, under test conditions, where authors themselves do not offer any help. The tool should speak for itself and the it should be obvious to the user how to achieve certain tasks.

A lot more hand holding is required for this to be of general use. Firstly, with the interface. How am i to choose the OG and the coordinates? I assume the coordinates are in base pairs, but when i open an e. coli genome and enter start coord: 1 and end coord: 3000000. I presume the program will show that section of the genome, but it doesn't. It seems to snap back to some strange number (maybe this is dictated by the OG values in the field above). I don't really understand.

- The GUI has been changed; we tried to make it more accurate and clear. Now we provide more detailed documentation, step-by-step tutorials (including video tutorials) to help users with the analysis (available at https://gcb.readthedocs.io/).
The program can be used in one of two main scenarios. In the first scenario, the main interest is the complexity profile, in which case the user can click on a certain region of the complexity profile, click the "Draw graph" button and the corresponding subgraph will be displayed in the Graph panel. In case the user is interested in a specific part of the genome, the coordinates can be found in the sources like NCBI, DOORS operon database, papers describing some

interesting locus, or using the Search panel based on gene product annotations. Both scenarios are illustrated in tutorials now.
The genome region for graph-based representation shouldn't be too long. It usually includes the region of interest (gene, operon, genome island) and its close neighbourhood. Too many nodes will result in long layout and rendering times. We set an upper limit on the possible length of the region for drawing the graph (100 kilobases).

The Search bar, doesn't explain what I am searching. Locus tags? i dont know, there should be a suggested value/auto complete dropbox for the search box. What values would work, what values would not work. I don't know.

- A hint was added to the Search bar. Searching is performed over product features of annotated genomes.

I played around with the program for quite some time, and I cannot figure out how to produce the figures they show in the manuscript.

- Graphs were rendered in Cytoscape based on JSON exported from the GCB (we mention this in the manuscript); we cover this step in tutorials now. Graphs may be exported in JPEG from the GCB, but no vector formats are supported for the export. The Cytoscape is used because it has a number of tools for arranging nodes and the ability to export images in vector format.

It took me a while to figure out where complexity plot is (it's not shown by default and seems to be hidden).

- The GUI has been modified, the complexity plot is immediately visible now.

What i am trying to point out, is that all the components are likely there, but I have no idea how to use it. Tool tips, more documentation, suggested values, worked examples, a demo, information in the manual are all absent.

- We have added tool tips to the interface and expanded the documentation.

For the website, The manual only explains the interface. It needs a step-by-step worked example to show people how to use it. Step-by-step, to the point where the manual says "Select X from the dropdown, then click button Y, ... " and so on.

- Step-by-step tutorials were added.

It is likely that users will want to use genomes other than the few provided - but judging from the manual the process seems quite involved to do your own data. can the authors simplify this approach somehow?

- We have consolidated the main logic into one script (gg.py), and added step-by-step tutorials to help with the analysis.
Orthogroup inference is the computationally intensive step, and often requires a computing cluster to execute. This step is mandatory in a standalone version usage. So, for now, we assume that users have basic command-line skills.

In terms of the manuscript.

This tool is used to detect hypervariable regions which in turn implies mobile genetic elements. One example given here is prophage regions in E. coli and Bacillus. I can see the figures are annotated with prediction from PHASTER But it is not clearly stated what are the differences between this approach and what PHASTER predicts. Doing a comprehensive comparison would show what the benefits and limitations of GCB. I think the authors need to take more time with these worked examples to show the utility of their tool.

- Regions of high variability are associated with prophages and mobile genetic elements, but are not limited to them. Variability hot-spots exist, which have no prophages or other mobility determinants (similar conclusions are made in [Oliveira et al., "The chromosomal organization of horizontal gene transfer in bacteria.", Nature communications, 2017]).
We have added a comparison of hot-spots identified by GCB with a curated dataset of genome islands to a new section called "Method applicability". The main purpose of a complexity analysis is to find variable genome regions. Other tools can identify prophages and mobile elements, but no tool can quantify local genome variability to find variability hot-spots that neither genome islands, nor prophages.

There is a question of scalabilty, how long would these graph traversals take if there was 1000 genomes for instance. The sample data seems quite small.

- We have measured a runtime for a 1000 E. coli genomes dataset, results are added to the manuscript text (Figure 7). The analysis, downstream to orthogroup inference, took nearly five hours for 1000 draft genomes with genome co-alignment step, and 20 minutes without co-alignment step (similar time will be needed for the analysis of 1000 complete genomes), on the laptop with i7 9750h, 16Gb RAM configuration. Orthogroup inference for 1000 genomes took five days, running in 50 threads on AMD EPYC 7502.
